# Depression in multiple sclerosis and associated factors

**DOI:** 10.1192/j.eurpsy.2025.863

**Published:** 2025-08-26

**Authors:** N. Ben Hamed, A. Aissa, A. Souissi, A. Labyedh, R. Jomli

**Affiliations:** 1 psychiatry A, razi hospital; 2 neurology department, LR18SP03 and Clinical Investigation Center Neurosciences and Mental Health - university hospital center Razi, Manouba, Tunisia

## Abstract

**Introduction:**

Multiple sclerosis (MS) is a chronic, immune-mediated disorder that affects the central nervous system, leading to a range of neurological impairments. Depression is a common comorbidity in MS, affecting up to 50% of patients over the course of the disease. This association not only worsens functional outcomes but also increases disability, reduces treatment adherence, and negatively impacts overall quality of life.

**Objectives:**

The aim of this study is to assess the prevalence of depression in patients with MS and identify its associated factors.

**Methods:**

A cross-sectional study was conducted in the neurology department of Razi University Hospital (Tunisia) between October 2023 and June 2024. Patients with a diagnosis of MS based on the 2017 McDonald criteria were recruited, excluding those with active disease relapses. Participants completed questionnaires covering sociodemographic data, medical history, clinical and radiological characteristicsand psychological symptoms. Disability was evaluated via the Expanded Disability Status Scale (EDSS). Depression, anxiety and stress were assessed using the DASS-21 scale. Insomnia was evaluated using the Pittsburgh Sleep Quality Index (PSQI). Data analysis was performed using SPSS version 26.

**Results:**

A total of 83 patients with MS were recruited, with ages ranging from 19 to 66 years. The study population had a predominantly female sex ratio of 3.4. The majority of participants (75.9%) were from urban areas, and 74.7% had a university-level education. Moreover, 49.1% were married, and 60.2% were employed. Regarding medical history, 40.3% had a comorbid condition, and 30.1% had a psychiatric history. The mean age at disease onset was 26 ± 10 years. First-line treatments (interferon, glatiramer acetate, teriflunomide, dimethyl fumarate) were prescribed to 27.7% of patients. Second-line treatments (natalizumab, ocrelizumab, fingolimod) were prescribed to 69.9% of patients.

In our study, the prevalence of depression was 52.6% according to the DASS-21 scale. In our population, 24.1% had severe depression, 18.1% had moderate depression, and 10.4%. Depression was significantly associated with employment status (p=0.05), socioeconomic status (p=0.02), personal organic history (p=0.01), personal psychiatric history (p=0.02), the presence of more than 10 lesions on MRI (p=0.01), disability status measured by the EDSS score (p=0.03), as well as anxiety, insomnia, and stress (p<0.001).

**Conclusions:**

The relationship between MS and depression is complex. Routine screening for depression during MS follow-ups is crucial. Effective management of mood disorders in MS patients can significantly improve their quality of life.

**Disclosure of Interest:**

None Declared

